# Bis(μ-pyridine-2,4-dicarboxyl­ato)-κ^3^ *N*,*O* ^2^:*O* ^2^;κ^3^ *O* ^2^:*N*,*O* ^2^-bis­[triaqua­magnesium(II)]

**DOI:** 10.1107/S1600536810030722

**Published:** 2010-08-11

**Authors:** Qing-Fu Zhang, Dan-Dan Han, Jian-Dong Pang, Ning-Ning Meng

**Affiliations:** a College of Chemistry and Chemical Engineering, Liaocheng University, Shandong 252059, People’s Republic of China

## Abstract

In the title centrosymmetric Mg^II^ complex, [Mg_2_(C_7_H_3_NO_4_)_2_(H_2_O)_6_], each Mg cation is *N*,*O*-chelated by a pyridine-2,4-dicarboxyl­ate dianion and is coordinated by three water mol­ecules. A carboxyl­ate O atom from the neighboring pyridine-2,4-dicarboxyl­ate dianion bridges the Mg cation to complete the MgNO_5_ distorted octa­hedral coordination geometry. The dinuclear complex mol­ecules are linked by inter­molecular O—H⋯O hydrogen bonding, forming a three-dimensional supra­molecular structure.

## Related literature

For the applications of Mg complexes, see: Davies *et al.* (2007 ▶); Dinca & Long (2005 ▶).
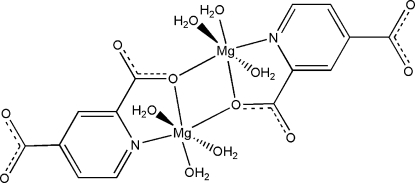


## Experimental

### 

#### Crystal data

[Mg_2_(C_7_H_3_NO_4_)_2_(H_2_O)_6_]
*M* *_r_* = 486.92
Orthorhombic, 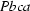

*a* = 7.9221 (8) Å
*b* = 12.0951 (12) Å
*c* = 20.2989 (18) Å
*V* = 1945.0 (3) Å^3^
*Z* = 4
Mo *K*α radiation
μ = 0.21 mm^−1^
*T* = 293 K
0.25 × 0.18 × 0.15 mm

#### Data collection

Bruker SMART CCD area-detector diffractometer
Absorption correction: multi-scan (*SADABS*; Sheldrick, 1996 ▶) *T*_min_ = 0.951, *T*_max_ = 0.970
8884 measured reflections
1719 independent reflections
1289 reflections with *I* > 2σ(*I*)
*R*_int_ = 0.042

#### Refinement

*R*[*F* ^2^ > 2σ(*F* ^2^)] = 0.040
*wR*(*F* ^2^) = 0.100
*S* = 1.06
1719 reflections
145 parameters
H-atom parameters constrained
Δρ_max_ = 0.36 e Å^−3^
Δρ_min_ = −0.30 e Å^−3^

### 

Data collection: *SMART* (Siemens, 1996 ▶); cell refinement: *SAINT* (Siemens, 1996 ▶); data reduction: *SAINT*; program(s) used to solve structure: *SHELXS97* (Sheldrick, 2008 ▶); program(s) used to refine structure: *SHELXL97* (Sheldrick, 2008 ▶); molecular graphics: *SHELXTL* (Sheldrick, 2008 ▶); software used to prepare material for publication: *SHELXTL*.

## Supplementary Material

Crystal structure: contains datablocks I, global. DOI: 10.1107/S1600536810030722/xu5007sup1.cif

Structure factors: contains datablocks I. DOI: 10.1107/S1600536810030722/xu5007Isup2.hkl

Additional supplementary materials:  crystallographic information; 3D view; checkCIF report

## Figures and Tables

**Table 1:** Selected bond lengths (Å)

Mg1—N1	2.2202 (19)
Mg1—O1	2.1011 (19)
Mg1—O1^i^	2.0613 (16)
Mg1—O5	2.0953 (19)
Mg1—O6	2.017 (2)
Mg1—O7	2.033 (2)

Symmetry code: (i) 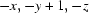.

**Table 2:** Hydrogen-bond geometry (Å, °)

*D*—H⋯*A*	*D*—H	H⋯*A*	*D*⋯*A*	*D*—H⋯*A*
O5—H5*A*⋯O4^ii^	0.87	1.80	2.668 (3)	172
O5—H5*B*⋯O2^iii^	0.86	2.12	2.834 (3)	140
O6—H6*A*⋯O3^iv^	0.87	1.73	2.576 (3)	163
O6—H6*B*⋯O5^v^	0.87	2.07	2.880 (2)	154
O7—H7*A*⋯O4^vi^	0.86	1.89	2.745 (2)	174
O7—H7*B*⋯O4^vii^	0.86	2.02	2.857 (3)	166

Symmetry codes: (ii) 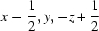; (iii) 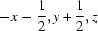; (iv) 
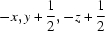
; (v) 
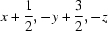
; (vi) 
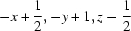
; (vii) 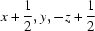.

**Fig. 1.**
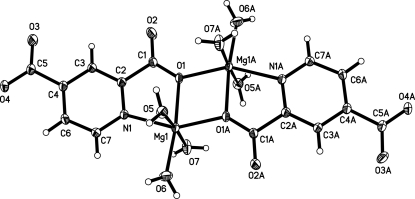
The molecular structure of the title compound, showing 30% probability displacement ellipsoids.

**Fig. 2.**
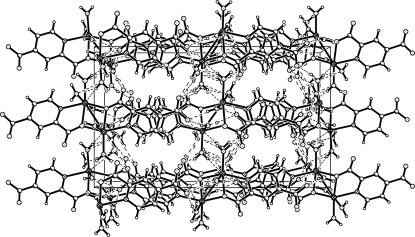
The packing diagram of the title complex.

## supplementary crystallographic information

### Comment

Magnesium(II) complexes have been extensively studied in recent year due to their wide application in synthesis of inorganic materials, catalysis of organic reactions and storage of hydrogen (Dinca & Long, 2005). In order to explore the relationship between these applications and their structures, a series of magnesium(II) complexes have been prepared and structural characterized (Davies *et al.*, 2007). To expand research in this area, here we report a dinuclear magnesium(II) complex.

As shown in Fig.1, each magnesium(II) ion in the title complex is coordinated by two carboxyl O atoms, one pyridyl N atom and three water molecules, forming a distorted octahedral geometry (axial angle, O7—Mg1—O5 = 173.33 (9)°). Interestingly, the two adjacent magnesium(II) ions are linked by two µ_2_-carboxylate O atoms to form a dinuclear strucutre.

In the crystal strucutre, these dimeric molecules are linked by intermolecular O—H···O H-bonding interactions into a three-dimensional framework (Fig.2).

### Experimental

A mixture of pyridine-2,4-dicarboxylic acid (16.7 mg, 0.10 mmol) and magnesium chloride hexahydrate (20.3 mg, 0.10 mmol) in 5 ml dimethyl acetamide (DMA) was heated to 373 K in a sealed 10 ml Teflon-lined reactor for 72 h. The mixture was allowed to cool to room temperature and the resulting block-shaped colorless crystals filtered from the reaction mixture. Yield, 72%.

### Refinement

The H atoms on water were located in a difference Fourier map and refined in riding mode with the fixed U_iso_(H) = 0.105 Å^2^. The aromatic H atoms were placed at calculated positions and were treated as riding on the parent C atoms with C—H = 0.93 Å and U_iso_(H) = 1.2U_eq_(C).

### Crystal data

**Table:**

[Mg_2_(C_7_H_3_NO_4_)_2_(H_2_O)_6_]	*F*(000) = 1008
*M**_r_* = 486.92	*D*_x_ = 1.663 Mg m^−^^3^
Orthorhombic, *P**b**c**a*	Mo *K*α radiation, λ = 0.71073 Å
Hall symbol: -P 2ac 2ab	Cell parameters from 2705 reflections
*a* = 7.9221 (8) Å	θ = 3.2–26.8°
*b* = 12.0951 (12) Å	µ = 0.21 mm^−^^1^
*c* = 20.2989 (18) Å	*T* = 293 K
*V* = 1945.0 (3) Å^3^	Block, colorless
*Z* = 4	0.25 × 0.18 × 0.15 mm

### Data collection

**Table:**

Bruker SMART CCD area-detector diffractometer	1719 independent reflections
Radiation source: fine-focus sealed tube	1289 reflections with *I* > 2σ(*I*)
graphite	*R*_int_ = 0.042
φ and ω scans	θ_max_ = 25.0°, θ_min_ = 2.0°
Absorption correction: multi-scan (*SADABS*; Sheldrick, 1996)	*h* = −5→9
*T*_min_ = 0.951, *T*_max_ = 0.970	*k* = −14→14
8884 measured reflections	*l* = −24→23

### Refinement

**Table:**

Refinement on *F*^2^	Primary atom site location: structure-invariant direct methods
Least-squares matrix: full	Secondary atom site location: difference Fourier map
*R*[*F*^2^ > 2σ(*F*^2^)] = 0.040	Hydrogen site location: inferred from neighbouring sites
*wR*(*F*^2^) = 0.100	H-atom parameters constrained
*S* = 1.06	*w* = 1/[σ^2^(*F*_o_^2^) + (0.0376*P*)^2^ + 1.7679*P*] where *P* = (*F*_o_^2^ + 2*F*_c_^2^)/3
1719 reflections	(Δ/σ)_max_ < 0.001
145 parameters	Δρ_max_ = 0.36 e Å^−^^3^
0 restraints	Δρ_min_ = −0.30 e Å^−^^3^

### Special details

**Table:**

Geometry. All e.s.d.'s (except the e.s.d. in the dihedral angle between two l.s. planes) are estimated using the full covariance matrix. The cell e.s.d.'s are taken into account individually in the estimation of e.s.d.'s in distances, angles and torsion angles; correlations between e.s.d.'s in cell parameters are only used when they are defined by crystal symmetry. An approximate (isotropic) treatment of cell e.s.d.'s is used for estimating e.s.d.'s involving l.s. planes.
Refinement. Refinement of *F*^2^ against ALL reflections. The weighted *R*-factor *wR* and goodness of fit *S* are based on *F*^2^, conventional *R*-factors *R* are based on *F*, with *F* set to zero for negative *F*^2^. The threshold expression of *F*^2^ > σ(*F*^2^) is used only for calculating *R*-factors(gt) *etc*. and is not relevant to the choice of reflections for refinement. *R*-factors based on *F*^2^ are statistically about twice as large as those based on *F*, and *R*- factors based on ALL data will be even larger.

### Fractional atomic coordinates and isotropic or equivalent isotropic displacement parameters (Å^2^)

**Table:**

	*x*	*y*	*z*	*U*_iso_*/*U*_eq_	
Mg1	0.07954 (11)	0.60169 (7)	0.04323 (3)	0.0240 (2)	
O1	−0.0075 (2)	0.43818 (14)	0.05141 (7)	0.0272 (4)	
O2	−0.0923 (4)	0.30663 (19)	0.12021 (10)	0.0760 (9)	
O3	−0.0102 (4)	0.3649 (2)	0.35781 (9)	0.0875 (11)	
H6A	0.1178	0.8068	0.0765	0.105*	
H5B	−0.1892	0.7246	0.0848	0.105*	
H5A	−0.2377	0.6193	0.0826	0.105*	
H7A	0.3503	0.5401	−0.0194	0.105*	
H7B	0.4054	0.5600	0.0457	0.105*	
H6B	0.2174	0.8016	0.0211	0.105*	
O4	0.1026 (2)	0.51899 (15)	0.39638 (8)	0.0364 (5)	
O5	−0.1604 (2)	0.66298 (14)	0.06670 (8)	0.0323 (5)	
O6	0.1539 (3)	0.76106 (16)	0.04659 (9)	0.0491 (6)	
O7	0.3209 (2)	0.56088 (17)	0.01937 (8)	0.0430 (5)	
N1	0.1126 (3)	0.56101 (17)	0.14911 (9)	0.0257 (5)	
C1	−0.0232 (4)	0.3941 (2)	0.10852 (11)	0.0325 (6)	
C2	0.0461 (3)	0.4620 (2)	0.16485 (11)	0.0261 (6)	
C3	0.0314 (3)	0.4258 (2)	0.22895 (11)	0.0302 (6)	
H3	−0.0135	0.3564	0.2378	0.036*	
C4	0.0838 (3)	0.4934 (2)	0.28011 (11)	0.0281 (6)	
C5	0.0576 (4)	0.4556 (2)	0.35037 (12)	0.0361 (7)	
C6	0.1530 (4)	0.5952 (2)	0.26430 (11)	0.0318 (6)	
H6	0.1902	0.6426	0.2973	0.038*	
C7	0.1660 (4)	0.6255 (2)	0.19836 (11)	0.0325 (6)	
H7	0.2139	0.6935	0.1881	0.039*	

### Atomic displacement parameters (Å^2^)

**Table:**

	*U*^11^	*U*^22^	*U*^33^	*U*^12^	*U*^13^	*U*^23^
Mg1	0.0328 (5)	0.0234 (5)	0.0157 (4)	−0.0004 (4)	0.0015 (3)	−0.0008 (3)
O1	0.0415 (11)	0.0250 (10)	0.0151 (8)	0.0001 (8)	−0.0028 (8)	0.0000 (7)
O2	0.151 (3)	0.0485 (15)	0.0289 (11)	−0.0543 (16)	−0.0205 (13)	0.0080 (10)
O3	0.176 (3)	0.0627 (17)	0.0241 (11)	−0.0682 (19)	0.0088 (14)	0.0028 (10)
O4	0.0497 (12)	0.0419 (12)	0.0176 (8)	−0.0064 (9)	−0.0043 (8)	−0.0016 (8)
O5	0.0379 (11)	0.0271 (10)	0.0320 (9)	0.0057 (8)	0.0049 (8)	−0.0031 (8)
O6	0.0683 (15)	0.0308 (11)	0.0482 (12)	−0.0158 (10)	0.0282 (11)	−0.0089 (9)
O7	0.0339 (11)	0.0693 (15)	0.0260 (9)	0.0104 (10)	−0.0014 (8)	−0.0103 (9)
N1	0.0348 (13)	0.0239 (12)	0.0185 (10)	−0.0021 (9)	−0.0003 (9)	0.0002 (8)
C1	0.0538 (18)	0.0229 (14)	0.0208 (13)	−0.0060 (13)	−0.0040 (12)	−0.0003 (11)
C2	0.0327 (15)	0.0238 (14)	0.0220 (12)	−0.0006 (11)	−0.0008 (11)	0.0010 (10)
C3	0.0445 (17)	0.0256 (14)	0.0205 (12)	−0.0074 (12)	−0.0023 (12)	0.0028 (10)
C4	0.0349 (15)	0.0301 (15)	0.0194 (12)	−0.0011 (12)	−0.0010 (11)	0.0007 (10)
C5	0.0526 (19)	0.0362 (17)	0.0193 (13)	−0.0074 (14)	−0.0014 (12)	0.0027 (12)
C6	0.0449 (16)	0.0308 (15)	0.0196 (12)	−0.0068 (13)	−0.0052 (12)	−0.0026 (10)
C7	0.0466 (17)	0.0275 (15)	0.0236 (13)	−0.0102 (13)	−0.0025 (12)	0.0009 (11)

### Geometric parameters (Å, °)

**Table:**

Mg1—N1	2.2202 (19)	O7—H7A	0.8581
Mg1—O1	2.1011 (19)	O7—H7B	0.8563
Mg1—O1^i^	2.0613 (16)	N1—C7	1.336 (3)
Mg1—O5	2.0953 (19)	N1—C2	1.347 (3)
Mg1—O6	2.017 (2)	C1—C2	1.511 (3)
Mg1—O7	2.033 (2)	C2—C3	1.378 (3)
O1—C1	1.282 (3)	C3—C4	1.385 (3)
O2—C1	1.215 (3)	C3—H3	0.9300
O3—C5	1.231 (3)	C4—C6	1.385 (4)
O4—C5	1.260 (3)	C4—C5	1.512 (3)
O5—H5B	0.8616	C6—C7	1.392 (3)
O5—H5A	0.8701	C6—H6	0.9300
O6—H6A	0.8688	C7—H7	0.9300
O6—H6B	0.8725		
			
O6—Mg1—O7	88.02 (9)	H6A—O6—H6B	104.2
O6—Mg1—O1^i^	109.63 (8)	Mg1—O7—H7A	123.0
O7—Mg1—O1^i^	88.93 (7)	Mg1—O7—H7B	126.1
O6—Mg1—O5	85.36 (8)	H7A—O7—H7B	110.8
O7—Mg1—O5	173.33 (9)	C7—N1—C2	117.7 (2)
O1^i^—Mg1—O5	92.50 (7)	C7—N1—Mg1	129.22 (17)
O6—Mg1—O1	173.16 (8)	C2—N1—Mg1	112.37 (15)
O7—Mg1—O1	95.68 (8)	O2—C1—O1	125.6 (2)
O1^i^—Mg1—O1	76.26 (7)	O2—C1—C2	119.3 (2)
O5—Mg1—O1	90.98 (8)	O1—C1—C2	115.0 (2)
O6—Mg1—N1	98.32 (8)	N1—C2—C3	122.7 (2)
O7—Mg1—N1	93.77 (8)	N1—C2—C1	116.5 (2)
O1^i^—Mg1—N1	152.00 (8)	C3—C2—C1	120.7 (2)
O5—Mg1—N1	88.02 (8)	C2—C3—C4	119.7 (2)
O1—Mg1—N1	75.74 (7)	C2—C3—H3	120.2
O6—Mg1—Mg1^i^	148.06 (7)	C4—C3—H3	120.2
O7—Mg1—Mg1^i^	92.97 (6)	C6—C4—C3	118.0 (2)
O1^i^—Mg1—Mg1^i^	38.56 (5)	C6—C4—C5	122.8 (2)
O5—Mg1—Mg1^i^	92.20 (6)	C3—C4—C5	119.2 (2)
O1—Mg1—Mg1^i^	37.70 (4)	O3—C5—O4	125.1 (2)
N1—Mg1—Mg1^i^	113.44 (7)	O3—C5—C4	116.5 (2)
C1—O1—Mg1^i^	135.94 (16)	O4—C5—C4	118.4 (2)
C1—O1—Mg1	119.66 (15)	C4—C6—C7	119.1 (2)
Mg1^i^—O1—Mg1	103.74 (7)	C4—C6—H6	120.5
Mg1—O5—H5B	130.0	C7—C6—H6	120.5
Mg1—O5—H5A	120.5	N1—C7—C6	122.9 (2)
H5B—O5—H5A	100.5	N1—C7—H7	118.6
Mg1—O6—H6A	122.4	C6—C7—H7	118.6
Mg1—O6—H6B	133.3		

Symmetry codes: (i) −*x*, −*y*+1, −*z*.

### Hydrogen-bond geometry (Å, °)

**Table:**

*D*—H···*A*	*D*—H	H···*A*	*D*···*A*	*D*—H···*A*
O5—H5A···O4^ii^	0.87	1.80	2.668 (3)	172.
O5—H5B···O2^iii^	0.86	2.12	2.834 (3)	140.
O6—H6A···O3^iv^	0.87	1.73	2.576 (3)	163.
O6—H6B···O5^v^	0.87	2.07	2.880 (2)	154.
O7—H7A···O4^vi^	0.86	1.89	2.745 (2)	174.
O7—H7B···O4^vii^	0.86	2.02	2.857 (3)	166.

Symmetry codes: (ii) *x*−1/2, *y*, −*z*+1/2; (iii) −*x*−1/2, *y*+1/2, *z*; (iv) −*x*, *y*+1/2, −*z*+1/2; (v) *x*+1/2, −*y*+3/2, −*z*; (vi) −*x*+1/2, −*y*+1, *z*−1/2; (vii) *x*+1/2, *y*, −*z*+1/2.

## References

[bb1] Davies, R. P., Less, R. J., Lickiss, P. D. & White, A. J. P. (2007). *Dalton Trans.* pp. 2528–2535. 10.1039/b705028c 17563788

[bb2] Dinca, M. & Long, J. R. (2005). *J. Am. Chem. Soc.* **127**, 9376–9377. 10.1021/ja0523082 15984858

[bb3] Sheldrick, G. M. (1996). *SADABS* University of Göttingen, Germany.

[bb4] Sheldrick, G. M. (2008). *Acta Cryst.* A**64**, 112–122. 10.1107/S0108767307043930 18156677

[bb5] Siemens. (1996). *SMART* and *SAINT* Siemens Analytical X-ray Instruments Inc., Madison, Wisconsin, USA.

